# Interaction of Adiponectin Genotypes and Insulin Resistance on the Occurrence of Taiwanese Metabolic Syndrome

**DOI:** 10.1155/2021/5570827

**Published:** 2021-04-30

**Authors:** Chun-Chieh Chen, Yu-Hsiang Wei, Chia-Chen Huang, Shin-Hui Hung, Zeng-Wei Wang, Ruey-Hong Wong

**Affiliations:** ^1^Department of Family and Community Medicine, Chung Shan Medical University, Taichung, Taiwan; ^2^School of Medicine, Chung Shan Medical University, Taichung, Taiwan; ^3^Department of Occupational Medicine, Chung Shan Medical University Hospital, Taichung, Taiwan; ^4^Department of Public Health, Chung Shan Medical University, Taichung, Taiwan

## Abstract

**Backgrounds:**

Adiponectin (apM1) may affect insulin sensitivity, and tumor necrosis factor (TNF-*α*) can inhibit the binding of insulin and insulin receptors. However, whether *apM1* and *TNF-α* genes influence the development of metabolic syndrome (MetS) preceded by insulin resistance is unclear. The current study examines the interactions between the *apM1* +45 genotypes, *TNF-α* -308 genotypes, and insulin resistance on the occurrence of MetS.

**Methods:**

A total of 329 community residents were recruited, and their personal characteristics were collected. Waist circumference and biochemical markers were examined for determining MetS. Genotypes were identified by the polymerase chain reaction.

**Results:**

After adjusting for the confounding effects, compared to *apM1* +45 GG and GT genotypes carriers with HOMR-IR less than 2.0, those carriers with HOMA-IR greater than 2.0 had an increased MetS risk (OR = 4.35, 95% CI 2.14-8.85). Further, *apM1* +45 TT carriers with HOMA-IR greater than 2.0 experienced a higher MetS risk (OR = 5.91, 95% CI 2.78-12.54). A significant interaction of the *apM1* +45 genotype and insulin resistance on the MetS development was observed (*P* = 0.04).

**Conclusion:**

Our data suggested that *apM1* +45 genotypes might modify the effect of insulin resistance on the development of Taiwanese MetS.

## 1. Introduction

Metabolic syndrome (MetS) is characterized by a cluster of metabolic disorders, including central obesity, dyslipidemia (high triglycerides and low high-density lipoprotein cholesterol (HDL-C)), hyperglycemia, and high blood pressure [1]. Importantly, insulin resistance is a critical feature of MetS [2] and is significantly associated with indexes of MetS.

Adipose tissue secretes adipokines, including adiponectin, tumor necrosis factor-*α* (TNF-*α*), interleukin-6 (IL-6), IL-18, and leptin, to regulate obesity. Dysregulation of the adipose tissue might increase the free fatty acid of the liver and muscles and contribute to insulin resistance and hyperlipidemia [3]. Adiponectin is also called AdipoQ and apM1. Unlike most hormones derived from the adipose tissue, the concentration of circulating apM1 is decreased in obese individuals [4]. Adiponectin stimulates fatty acid oxidation and directly affects insulin sensitivity [5]. Further, it has been suggested that low apM1 concentration is an independent risk factor of insulin resistance [6] and type 2 diabetes (T2D) [7]. The association between circulating apM1 levels and the indexes of MetS has also been reported [8]. The human *apM1* gene is located on chromosome 3q27 established by genome-wide scans [9]. A single-nucleotide polymorphism (SNP, rs2241766) is located in exon 2, which can affect gene transcription and its secretion, and the proportion of G allele is 20.2%-38.5% in Chinese people [10]. However, the role of *apM1* +45 polymorphism on the development of Taiwanese MetS preceded by insulin resistance is unclear.

Moreover, TNF-*α* is a potent inhibitor of insulin signalling [11, 12]. When TNF-*α* binds with receptors, nuclear factor-*κ*B will be evoked and result in an inflammatory reaction [12]. Besides, TNF-*α* may influence the fat metabolism [13] and is positively correlated with circulating triglycerides (TG) [14], obesity, and insulin resistance [15]. Thus, *TNF-α* has been suggested to play an important role in MetS. The human *TNF-α* gene is located on chromosome 6p21.3, the area between human leukocyte antigen complex (*HLA* complex) class I and class II region [16]. A SNP (G ⟶ A, guanine to adenine; rs1800629) at position -308 of the *TNF-α* promoter region was identified [16]. Under in vitro stimulation, the *TNF-α* -308 A allele increased the TNF-*α* expression [17]. Therefore, the structural variation of this gene may affect the development of MetS preceded by insulin resistance.

Though *apM1* and *TNF-α* were considered to play critical roles on the development of insulin resistance, few studies based on general population have been designed to investigate this relation. Therefore, the current community-based study tests the interactions between the *apM1* genotypes, *TNF-α* genotypes, and insulin resistance on the occurrence of Taiwanese MetS.

## 2. Materials and Methods

### 2.1. Study Population and Epidemiological Data

A community-based health examination in central Taiwan (Taichung city and Nantou County) was carried out from September 2003 to July 2006. The study complied with the Helsinki declaration and was approved by the institutional review board of the Chung Shan Medical University Hospital, Taichung, Taiwan (CS09106). A total of 333 Taiwanese adults were recruited. Four subjects who self-reported having chronic renal disease were excluded. None of the subjects were previously diagnosed with cerebrovascular disease, peripheral vascular disease, adrenogenital syndrome, or primary aldosteronism. All subjects gave informed consent prior to inclusion in the study and agreed to all processes during this study.

### 2.2. Risk Factors

All subjects were asked to complete a structured questionnaire, and the questions included demographic characteristics, cigarette smoking, alcohol consumption, and personal medical history. Subjects were also asked to determine the amount, frequency, and duration of cigarette smoking and alcohol drinking. Because alcohol consumption is low among Taiwanese individuals, habitual alcohol drinking was defined as alcohol consumption on at least one occasion weekly and more than 80 grams of alcohol weekly, as in our previous study [18].

Health examinations were carried out in the morning or afternoon. Subjects were instructed to fast for at least 8 hours prior to examinations. Fasting plasma glucose was measured with hexokinase-glucose dehydrogenase by Olympus AU-5000. Total cholesterol, TG, HDL-C, and low-density lipoprotein-cholesterol (LDL-C) were measured using an automatic machine (Hitachi 7250; Hitachi Medical Corp., Japan) at the central laboratory of the hospital. In the present study, diabetes mellitus (DM) was defined as subjects who self-reported having DM, have fasting plasma glucose ≥ 126 mg/dL, or were taking insulin/oral hypoglycemic agents. Hyperlipidemia was defined as the presence of any one of the following conditions: subjects with fasting plasma total cholesterol ≥ 240 mg/dL, TG ≥ 200 mg/dL, LDL‐C ≥ 160 mg/dL, HDL‐C < 40 mg/dL for men and < 50 mg/dL for women, a total cholesterol/HDL‐C ratio ≥ 5, or taking hypolipidemic drugs [19]. In addition, 19 subjects with hypertension regularly took antihypertensive drugs. These subjects were categorized as hypertensive cases. Therefore, in this study, hypertension was defined as an average systolic blood pressure (SBP) ≥ 140 mmHg, an average diastolic blood pressure (DBP) ≥ 90 mmHg, and/or a history of hypertension that is regularly treated with antihypertensive drugs.

### 2.3. Definition of Taiwanese MetS

Metabolic syndrome in Asian populations is defined as individuals meeting at least three criteria as follows: (1) waist circumference ≥ 90 cm for men and ≥ 80 cm for women, (2) fasting TG level ≥ 150 mg/dL, (3) HDL‐C level < 40 mg/dL for men and < 50 mg/dL for women, (4) SBP ≥ 130 mmHg or DBP ≥ 85 mmHg, and (5) fasting plasma glucose ≥ 100 mg/dL [20].

The current study found 134 subjects (40.7%) with an abnormal waist circumference, 179 subjects (54.4%) with a fasting TG level ≥ 150 mg/dL, 122 subjects (37.0%) with an abnormal HDL-C level, 175 subjects (53.2%) with abnormal blood pressure, and 92 subjects (28.0%) with a fasting plasma glucose ≥ 100 mg/dL, respectively.

### 2.4. Evaluation of Homeostasis Model Assessment-Insulin Resistance (HOMA-IR), Triglyceride Glucose (TyG) Index, and TG/HDL-C Ratio

Insulin resistance was calculated by HOMA-IR, TyG index, and TG/HDL-C ratio, respectively. An index of HOMA-IR was calculated by using the following formula: HOMA‐IR index = (fasting plasma glucose × fasting insulin)/22.5. In a study with 2,649 Chinese subjects [21], the cut-off value of HOMA-IR to identify DM was 1.97 as determined by the receiver-operating characteristic (ROC) curve and 2.03 in the 90th percentile of subjects with normal glucose tolerance. Because most Taiwanese ancestors emigrated from China, we also defined the HOMA-IR cut-off value as 2.0 in the current study, as a previous study conducted in Taiwanese population [22]. The sensitivity, specificity, and area under the ROC curve (AUC) were 0.50, 0.80, and 0.75 (95% confidence interval (95% CI) 0.70-0.80), respectively, using the cut-off value of 2.0 to discriminate MetS from non-MetS in our subjects. The TyG index was calculated as follow: TyG index = ln [fasting TG (mg/dL) × fasting plasma glucose (mg/dL)/2]. The cut-off value of 9.0 was according to the median of TyG index in our subjects without MetS and with highest Youden index. The sensitivity, specificity, and AUC were 0.84, 0.77, and 0.88 (95% CI 0.84-0.92), respectively, using the best cut-off value to discriminate MetS from non-MetS in our subjects. The TG/HDL-C ratio was further calculated after dividing TG levels by HDL-C levels. The sensitivity, specificity, and AUC were 0.88, 0.71, and 0.87 (95% CI 0.83-0.91), respectively, using the cut-off value of 4.2 with highest Youden index to discriminate MetS from non-MetS in our subjects.

### 2.5. Determination of Genetic Polymorphisms

Polymerase chain reaction-restriction fragment length polymorphism (PCR-RFLP) was used to distinguish the variation of SNP 45 (T ⟶ G, thymine to guanine) in *apM1*. Primers used for the amplification of *apM1* gene were 5′-GCA GCT CCT AGA AGT AGA CTC TG-3′ and 5′-CCA AAT CAC TTC AGG TTG CTT-3′. One half microliter of DNA was added to a PCR buffer containing 200 ng of primers, 1.5 mM MgCl_2_, 0.2 mM of deoxyribonucleoside triphosphate, 50 mM KCl, 10 mM Tris-HCl (pH 8.3), and 0.1% of bovine serum albumin in a final volume of 50 *μ*L. Amplification was carried out under conditions that the initial incubation of 4 min at 94°C, followed by 35 cycles of denaturing step at 94°C for 30 sec, annealing at 60°C for 30 sec, and extension at 72°C for 40 sec. The reaction was terminated after a final extension of 5 min at 72°C. The PCR products were then digested with *Bsp*HI. Digestion products were visualized on a 4% agarose gel stained with ethidium bromide. Homozygous TT individuals had a product fragment of 479 bp, homozygous GG individuals had two product fragments of 313 and 166 bp, and heterozygous TG individuals had all three fragments.

Similar to the *apM1* +45 polymorphism, the detection of *TNF-α* -308 genotypes was conducted by PCR-RFLP. Primers used for the amplification of the *TNF-α* gene were 5′-AGG CAA TAG GTT TTG AGG GGC CAT-3′ and 5′-TCC TCC CTG CTC CGA TTC CG-3′. Amplification was carried out under conditions that the initial incubation of 5 min at 94°C, followed by denaturing step at 94°C for 30 sec, annealing at 60°C for 35 sec, and extension at 72°C for 60 sec. All steps were repeated for a total of 30 cycles. The reaction was terminated after a final extension of 5 min at 72°C. The PCR products were then digested with *Nco*I. Homozygous AA individuals had a product fragment of 107 bp, homozygous GG individuals had two product fragments of 87 and 20 bp, and heterozygous AG individuals had all three fragments.

### 2.6. Statistical Analysis

Comparisons between both groups with and without MetS for demographic characteristics, lifestyles, medical histories, anthropometric indexes, biochemical tests, genotypes of *apM1* +45 and *TNF-α* -308 were made using Student's *t*-test for continuous variables and *χ*^2^-test or Fisher's exact test for discrete variables. Further, goodness-of-fit *χ*^2^-test was used to test the genotypes for the Hardy-Weinberg equilibrium. Subsequently, a multiple logistic regression model was applied to obtain the odds ratio (OR) and 95% CI for each variable. Dominant and recessive models of inheritance were also used to evaluate the associations between genetic variants and MetS. Interaction was further assessed using the likelihood ratio test to calculate the *χ*^2^ and *P* values. Likelihood ratio *χ*^2^ test was also utilized to test the interactions between genotypes and insulin resistance indicators with respect to the risk of MetS. In the test for interaction, the logistic regression model with only main effects was compared to that with both main effect terms and interaction term. All data were analyzed using SAS 9.4 (SAS, Inc., Cary, NC, USA). Statistical tests that were two-tailed with *P* < 0.05 were considered statistically significant.

## 3. Results

In total, 329 adult community residents in central Taiwan were recruited in this study, and 121 (36.8%) were identified as having MetS (Table 1). The mean age of all subjects was 47.1 ± 0.8 (standard deviation) years and 239 (72.6%) subjects were male. The age and sex did not significantly differ between the groups with and without MetS. Among the study subjects, there were 64 (19.5%) individuals with DM, 6 (1.8%) individuals with heart disease, 115 (35.0%) individuals with hypertension, and 196 (59.6%) individuals with hyperlipidemia. Higher proportions of DM (35.5% versus 10.1%, *P* < 0.001, *χ*^2^-test), hypertension (59.5% versus 20.7%, *P* < 0.001), and hyperlipidemia (93.4% versus 39.9%, *P* < 0.001) were observed in the group with MetS than those in the group without MetS, respectively. Abnormal waist circumference was also prevalent in the group with MetS than that in the group without MetS (67.8% versus 25.0%, *P* < 0.001). As expected, SBP (140.1 versus 124.0 mmHg, *P* < 0.001, *t*-test), DBP (88.7 versus 78.8 mmHg, *P* < 0.001), serum TG (343.3 versus 145.7 mg/dL, *P* < 0.001), insulin levels (26.5 versus 12.0 *μ*U/mL, *P* < 0.001), fasting plasma glucose (127.3 versus 97.7 mg/dL, *P* < 0.001), HOMA-IR (3.9 versus 1.3, *P* < 0.001), TyG index (9.7 versus 8.7, *P* < 0.001), and TG/HDL-C ratio (9.2 versus 3.2, *P* < 0.001) were also significantly higher in the group with MetS than those in the group without MetS, respectively. Subjects with MetS also had lower HDL-C levels than those without MetS (40.0 versus 50.6 mg/dL, *P* < 0.001). However, there were no significant differences between the groups with and without MetS in the terms of education level, proportions of cigarette smoking and alcohol consumption, and LDL-C levels, respectively.

The associations of *apM1* +45 and *TNF-α* -308 genotypes with the occurrence of MetS in the study subjects are presented in Table 2. Frequencies of *apM1* +45 T and G allele were 73.6% and 26.4% in the subjects with MetS, respectively, while the frequencies were 69.5% and 30.5% in those without MetS, respectively. Frequencies of *TNF-α* -308 A and G alleles were 11.2% and 88.8% in the subjects with MetS, respectively, while the frequencies were 10.3% and 89.7% in those without MetS, respectively. In the MetS-free subjects, both of *apM1* +45 (*P* = 0.90) and *TNF-a* -308 (*P* = 0.56) polymorphisms conformed to the Hardy-Weinberg equilibrium. After adjusting for the effects of age and gender, subjects with *apM1* +45 TT genotype were not at significantly higher risk of MetS compared to those with *apM1* +45 TG/GG genotypes in the dominant model (OR = 1.34, 95% CI 0.85-2.11). Similarly, the results did not show any significant associations between *TNF-α* -308 genotypes and alleles with the MetS occurrence. The genetic effects of *apM1* +45 and *TNF-α* -308 on Taiwanese MetS were larger in the dominant models than those in the recessive models, respectively. Further, since the numbers of *apM1* +45 GG genotype and *TNF-α* -308 AA genotype were small, those were further combined with *apM1* +45 TG genotype and *TNF-α* -308 AG genotype, respectively, to increase the statistical power.

Subsequently, the analysis for interactions of *apM1* +45 genotypes, *TNF-α* -308 genotypes with fasting plasma glucose, and HOMA-IR on the MetS occurrence is shown in Table 3. After adjusting for the effects of age and gender, the results revealed *apM1* +45 TT carriers with fasting plasma glucose greater than 100 mg/dL (OR = 7.59, 95% CI 3.69-15.63), and *apM1* +45 GG/GT carriers with fasting plasma glucose greater than 100 mg/dL (OR = 5.86, 95% CI 2.79-12.32) had significantly higher risks of MetS than *apM1*+45 GG/GT carriers with fasting plasma glucose less than 100 mg/dL. Similar combined effects of *TNF-α* -308 genotypes and fasting plasma glucose on the MetS risk were also observed. However, the interaction of *apM1* +45 genotypes and fasting plasma glucose on the MetS occurrence did not reach statistical significance. Next, the results also revealed *apM1* +45 TT carriers with HOMA-IR greater than 2.0 had a 5.91-fold (95% CI 2.78-12.54) greater risk, and *apM1* GG/GT carriers with HOMA-IR greater than 2.0 had a 4.35-fold (95% CI 2.14-8.85) greater risk of developing MetS as compared to *apM1* +45 GG/GT carriers with HOMA-IR less than 2.0, respectively. In particular, a significant interaction between *apM1* +45 genotypes and HOMA-IR on the MetS occurrence was observed (*P* = 0.04). However, the interaction between *TNF-α* -308 genotypes and HOMA-IR on the occurrence of MetS was not statistically significant.

Finally, the interactions of *apM1* +45 genotypes, *TNF-α* -308 genotypes with TyG index, and TG/HDL-C ratio on the MetS occurrence were also evaluated (Table 4). The results revealed *apM1* +45 TT carriers with TyG index greater than 9.0 (OR = 22.78, 95% CI 9.56-54.30), and *apM1* +45 GG/GT carriers with TyG index greater than 9.0 (OR = 19.20, 95% CI 7.96-46.31) had significantly higher risks of MetS than *apM1* +45 GG/GT carriers with TyG index less than 9.0. Similar effects were also revealed between *apM1* +45 genotypes and TG/HDL-C ratio on the risk of MetS occurrence. However, the interactions of *apM1* +45 genotypes with TyG index and TG/HDL-C ratio on the MetS occurrence did not reach statistical significance, respectively. When *apM1* +45 genotypes were replaced by *TNF-α* -308 genotypes in the following analyses, the interactions of *TNF-α* -308 genotypes with TyG index and TG/HDL-C ratio on the occurrence of MetS did not reach statistical significance, either.

## 4. Discussion

In the current study, a significant interaction between *apM1* +45 genotypes and HOMA-IR on the MetS development was observed.

Individuals with MetS had been reported to experience a lower circulating apM1 concentration than those without MetS [23]. However, the role of *apM1* +45 polymorphism on the development of MetS is unclear. It has been indicated the genetic polymorphism of *apM1* +45 could affect circulating apM1 concentrations by influencing pre-mRNA splicing or mRNA stability and might be related to another functional locus via linkage disequilibrium [24]. A genome-wide linkage scan of plasma apM1 levels was performed in 569 nondiabetic participants from the Amish families [25]. This research also showed the *apM1* +45 experienced moderate relation in a dosage-dependent manner with apM1 levels. Therefore, the speculation that the association of *apM1* +45 polymorphism with specific metabolic disorders should be reasonable. Previously, Li et al. [26] found the T2D group exhibited a higher *apM1* +45 G allele frequency compared with the normal glucose tolerance group and lower plasma apM1 concentrations in the G allele carriers than those with the TT genotype in Uygurs of the Xinjiang region, China. In a meta-analysis study [27], a total of 2,819 obese subjects and 3,024 controls from 18 case-control studies were included. The results observed the *apM1* +45 GG genotype increased obesity risk in the Chinese studies. On the contrary, Yang et al. [28] found a correlation between *apM1* +45 TT genotype and insulin resistance in Chinese population. An earlier study also showed that *apM1* +45 G allele was associated with a reduction of 1.12 kg/m^2^ in body mass index in Taiwan population [29]. However, no independent association of *apM1* +45 genotypes with Taiwanese MetS development was found in the present study. No association was also observed between *apM1* +45 polymorphism and T2D in a case-control study with 149 T2D patients and 139 healthy conducted in Taiwan [30]. Taken the above mentioned together, it should be noted that the inconsistent associations in different studies may not only be due to subjects' races. Discrepancies among association studies may be also explained by sample size, or even in the linkage disequilibrium between the genetic variants detected in the *apM1* gene in different populations. More importantly, metabolic disorders are not only determined by apM1 but also are influenced by other multiple genetic and environmental factors. However, the interaction between *apM1* +45 variants and environmental factors on metabolic disorders did not be examined in most studies.

The relationship of insulin resistance and MetS has been proposed [2]. As expected, the values of insulin resistance indicators were higher in our MetS subjects than those in non-MetS subjects, including fasting plasma glucose, TyG, TG/HDL-C ratio, and HOMA-IR. It is known hyperinsulinemia precedes the development of many aspects of MetS, including hypertension, hyperlipidemia, and the development of T2D [2]. Hyperinsulinemia also increases liver production of very-low-density lipoprotein to increase serum TG, while simultaneously reducing HDL-C production [5, 31]. Taken together, evidence suggests insulin resistance is the primary factor responsible for MetS. In keeping with this speculation, we found that individuals with greater insulin resistance had a significantly higher risk of MetS, regardless of genotypes. Interestingly, a significant interaction of *apM1* +45 genotypes and HOMA-IR on the MetS development was further observed. Under our observed interaction, insulin resistance is aggravated when apM1 secretion decreases, while insulin resistance will further develop by decreasing utilization of glucose in the liver and skeletal muscle, thus leading to the occurrence of MetS [5]. Besides, it is also possible *apM1* +45 variants interact with environmental factors to influence MetS susceptibility via an effect not on circulating apM1 levels but rather on the apM1 level within adipose tissue. However, our observations and inferences need to be further confirmed.

In addition to HOMA-IR in the present study, the effect of insulin resistance on the MetS occurrence was also evaluated by using TyG index and TG/HDL-C ratio, respectively. As the results from the recent studies [32, 33], our data indicated TyG index and TG/HDL-C ratio were significant correlation factors of MetS. Some potential mechanisms might help explain such a result. It is clearly known that apM1 increases HDL and decreases TG [6]. The higher TG levels can increase free fatty acids and increase free fatty acids from adipose to nonadipose tissue, and then may induce insulin resistance [34]. Nevertheless, there were no interactions between *apM1* +45 genotypes, *TNF-α* -308 genotypes, with TyG index, and TG/HDL-C ratio on the development of MetS in our study. Even compared to HOMA-IR, they showed a better ability to distinguish MetS from our subjects. This further implies that *apM1* +45 variants mediated the effect of insulin resistant on MetS in our subjects through the regulation of glucose homeostasis rather than the modification of lipid metabolism.

Besides, TNF-*α* is suggested to be associated with obesity-related insulin resistance [15]. The expression of TNF-*α* might increase when substituting guanine by adenosine at *TNF-α* -308, and this might affect the development of MetS. However, an earlier meta-analysis enrolled 7,611 T2D patients and 6,944 controls (including Caucasian and Asian) from 18 studies; the conclusions proposed that *TNF-α* -308 genotypes had no relation to T2D [35]. In our study, *TNF-α* -308 A allele carriers did not show a higher risk on the development of MetS, and there is no interaction between *TNF-α* -308 genotypes and insulin resistance on the development of MetS. This might be also due to a small sample size restricting the statistical power of slight increase in risk.

Using the same criteria as the present study, another community-based health examination in southwestern Taiwan found the MetS prevalence in adults was 38.6% [36]. In the present study, the prevalence of MetS was 36.8%. In addition, males were suggested to have lower apM1 concentrations than females [8, 37]. Therefore, lower apM1 concentrations might explain the variations on the MetS development between both genders. The present study found subjects with MetS had a higher proportion of hypertension. Such a result may be associated with insulin resistance. It was found insulin is a vasodilator [38]. As mentioned above, insulin resistance is aggravated when apM1 secretion decreases [5]. Blood insulin might contribute to the synthesis of collagen protein and stimulate hyperplasia and hypertrophy of smooth muscle cells of the blood vessels [38], thus resulting in a higher risk of hypertension in subjects with MetS. This result further suggests that MetS could be an indicator of cardiovascular diseases. The present study also found subjects with MetS had a higher proportion of hyperglycemia. However, hemoglobin A1c (HbA1c) levels were not measured in our subjects. Higher cost was a determinant in recommendation of HbA1c as a screening test. However, in a comparison of fasting plasma glucose and HbAlc for diagnosing Taiwanese DM, TG, and HDL levels did not differ in subgroups diagnosed using fasting plasma glucose alone versus diagnosed by fasting glucose and HbA1c [39]. A good agreement between HbA1c and fasting plasma glucose in identifying individuals with MetS was also often proposed [40, 41]. Thus, fasting plasma glucose was used to detect the association with MetS in the present study.

The present study tests the interactions between the *apM1* +45 genotypes, TNF-*α* -308 genotypes, and insulin resistance on the occurrence of Taiwanese MetS. Even if there is biological evidence, it cannot be confused that the overall result is only statistically relevant. On the other hand, given a type I (*α*) error of 0.05, type II (*β*) error of 0.2, the proportion of *apM1* +45 TT genotype among MetS-free subjects of 0.42, detectable OR of 1.8, and the MetS prevalence of 33%, and the minimum sample size required for the MetS group is 138. In the current community-based study, 121 (36.8%) cases of MetS were identified. Certainly, smaller sample size is an obvious shortcoming of the current study. Our results had limited inferential capability. Inevitably, MetS are related to certain lifestyle, such as unhealthy diet and physical inactivity [1]. However, data on dietary pattern and physical activity were not available in the current community-based study. There is concern that estimates of dietary pattern and physical activity derived from self-report instruments are never free of errors, and most subjects in the communities have difficulties in estimating the specific dietary pattern and physical activity. In addition, no ancestry markers were detected in this study. However, all our participants were of the same race, which aided in reducing possible bias arising from different ethnic groups. The subjects volunteering to participate in this community-based health examination might have been healthier, and subject selection might have led to biased results. The present study did not detect individual circulating apM1 concentrations, and thus, we could not infer the cause-effect relation between the development of MetS, circulating apM1 levels, and associated factors.

In conclusion, our data suggested that *apM1* +45 variants might modify the effect of insulin resistance on the development of Taiwanese MetS. Larger studies would be necessary to provide further evidence regarding this finding.

## Figures and Tables

**Table 1:** The distributions of demographic characteristics, lifestyles, medical histories, anthropometric indexes, and biochemical tests in the study subjects.

Variables	With MetS	Without MetS	All
	*n* = 121	*n* = 208	*n* = 329
Age (years)	48.5 ± 1.2^#^	46.3 ± 1.0	47.1 ± 0.8
Gender: male	95 (78.5%)	144 (69.2%)	239 (72.6%)
Education			
Below senior high school	54 (44.6%)	93 (44.7%)	147 (44.7%)
Senior high school	52 (43.0%)	82 (39.4%)	134 (40.7%)
Above college	15 (12.4%)	33 (15.9%)	48 (14.6%)
Smoking	65 (53.7%)	95 (45.7%)	160 (48.6%)
Alcohol drinking	40 (33.1%)	51 (24.5%)	91 (27.7%)
Diabetes mellitus	43 (35.5%)	21 (10.1%)^∗^	64 (19.5%)
Heart disease	2 (1.0%)	4 (1.9%)	6 (1.8%)
Hypertension	72 (59.5%)	43 (20.7%)^∗^	115 (35.0%)
Hyperlipidemia	113 (93.4%)	83 (39.9%)^∗^	196 (59.6%)
SBP (mmHg)	140.1 ± 1.8	124.0 ± 1.3^∗^	129.9 ± 1.1
DBP (mmHg)	88.7 ± 1.2	78.8 ± 0.8^∗^	82.4 ± 0.7
Abnormal waist circumference	82 (67.8%)	52 (25.0%)^∗^	134 (40.7%)
Serum TG (mg/dL)	343.3 ± 26.3	145.7 ± 7.3^∗^	218.4 ± 11.9
HDL-C (mg/dL)	40.0 ± 0.8	50.6 ± 0.8^∗^	46.7 ± 0.6
LDL-C (mg/dL)	113.6 ± 3.4	116.9 ± 2.5	115.7 ± 2.0
Insulin (*μ*U/mL)	26.5 ± 2.5	12.0 ± 0.8^∗^	17.3 ± 1.1
Fasting plasma glucose (mg/dL)	127.3 ± 5.1	97.7 ± 2.8^∗^	108.6 ± 2.7
≥ 100 mg/dL	76 (62.8%)	52 (25.0%)^∗^	128 (38.9%)
HOMA-IR	3.9 ± 0.4	1.3 ± 0.1^∗^	2.2 ± 0.2
≥ 2.0	60 (49.6%)	42 (20.2%)^∗^	102 (31.0%)
TyG index	9.7 ± 0.1	8.7 ± 0.04^∗^	9.0 ± 0.1
≥ 9.0	102 (84.3%)	49 (23.6%)^∗^	151 (45.9%)
TG/HDL-C ratio	9.2 ± 8.5	3.2 ± 2.8^∗^	5.4 ± 6.3
≥ 4.2	100 (82.6%)	44 (21.2%)	144 (43.8%)

^#^Mean ± standard deviation. SBP: systolic blood pressure; DBP: diastolic blood pressure; TG: thyroglobulin; HDL-C: high-density lipoprotein cholesterol; LDL-C: low-density lipoprotein cholesterol; HOMA-IR: homeostatic model assessment for insulin resistance; TyG: triglyceride glucose. ^∗^The *P* value is < 0.01, Student's *t*-test for continuous variables and *χ*^2^-test or Fisher's exact test for discrete variables.

**Table 2:** Association of *apM1* +45 and *TNF-α* -308 genotypes with the development of Taiwanese metabolic syndrome.

Variables	With MetS	Without MetS	OR (95% CI)^#^
	*n* = 121	*n* = 208	
*apM1* +45 genotypes			
TT	67 (55.4%)	100 (48.1%)	1.23 (0.54-2.84)
TG	44 (36.3%)	89 (42.8%)	0.91 (0.39-2.13)
GG	10 (8.3%)	19 (9.1%)	1.00 (ref.)
Dominant model			
TT	67 (55.4%)	100 (48.1%)	1.34 (0.85-2.11)
TG/GG	54 (44.6%)	108 (51.9%)	1.00 (ref.)
Recessive model			
TT/TG	111 (91.7%)	189 (90.9%)	1.08 (0.48-2.42)
GG	10 (8.3%)	19 (9.1%)	1.00 (ref.)
T allele	178 (73.6%)	289 (69.5%)	1.21 (0.85-1.73)
G allele	64 (26.4%)	127 (30.5%)	1.00 (ref.)
*TNF-α* -308 genotypes			
AA	1 (0.8%)	3 (1.4%)	0.69 (0.07-6.97)
AG	25 (20.7%)	37 (17.8%)	1.17 (0.66-2.09)
GG	95 (78.5%)	168 (80.8%)	1.00 (ref.)
Dominant model			
AA/AG	26 (21.5%)	40 (19.2%)	1.49 (0.15-14.94)
GG	95 (78.5%)	168 (80.8%)	1.00 (ref.)
Recessive model			
AA	1 (0.8%)	3 (1.4%)	0.67 (0.07-6.75)
AG/GG	120 (99.2%)	205 (98.6%)	1.00 (ref.)
A allele	27 (11.2%)	43 (10.3%)	1.09 (0.65-1.83)
G allele	215 (88.8%)	373 (89.7%)	1.00 (ref.)

^#^Adjusted the effects of age and gender. OR: odds ratio; CI: confidence interval; ref.: reference.

**Table 3:** Interactions of *apM1* +45, *TNF-α* -308 genotypes with fasting plasma glucose, and HOMA-IR on the development of Taiwanese metabolic syndrome.

Variables	*apM1* +45 genotypes				*TNF-α* -308 genotypes			
	TT		GG/GT		AA/AG		GG	
	With/without MetS^#^ (OR (95% CI))^†^		With/without MetS^#^ (OR (95% CI))^†^		With/without MetS^#^ (OR (95% CI))^†^		With/without MetS^#^ (OR (95% CI))^†^	
Fasting plasma glucose								
≥ 100 mg/dL	44/28	7.59 (3.69-15.63)^∗^	32/24	5.86 (2.79-12.32)^∗^	19/13	6.47 (2.75-15.24)^∗^	57/39	5.99 (3.31-10.82)^∗^
< 100 mg/dL	23/72	1.20 (0.62-2.35)	22/84	1.00 (ref.)	7/27	0.95 (0.38-2.37)	38/129	1.00 (ref.)
Test for interaction	*χ* ^2^ = 0.02 (1 df); *P* = 0.88				*χ* ^2^ = 0.04 (1 df); *P* = 0.84			
HOMA-IR								
≥ 2.0	30/18	5.91 (2.78-12.54)^∗^	30/24	4.35 (2.14-8.85)^∗^	10/6	4.35 (1.47-12.87)^∗^	50/36	4.25 (2.27-7.41)^∗^
< 2.0	37/82	1.58 (0.86-2.88)	24/84	1.00 (ref.)	16/34	1.47 (0.73-2.95)	45/132	1.00 (ref.)
Test for interaction	*χ* ^2^ = 4.40 (1 df); *P* = 0.04				*χ* ^2^ = 0.04 (1 df); *P* = 0.84			

^#^Indicated the numbers of subjects with and without MetS, respectively. ^†^Adjusted the effects of age and gender. OR: odds ratio; CI: confidence interval; HOMA-IR: homeostatic model assessment for insulin resistance; ref.: reference. ^∗^The *P* value is < 0.01.

**Table 4:** Interactions of *apM1* +45 genotypes, *TNF-α* -308 genotypes with TyG index, and TG/HDL-C ratio on the development of Taiwanese metabolic syndrome.

Variables	*apM1* +45 genotypes				*TNF-α* -308 genotypes			
	TT		GG/GT		AA/AG		GG	
	With/without MetS^#^ (OR (95% CI))^†^		With/without MetS^#^ (OR (95% CI))^†^		With/without MetS^#^ (OR (95% CI))^†^		With/without MetS^#^ (OR (95% CI))^†^	
TyG index								
≥ 9.0	56/25	22.78 (9.56-54.30)^∗^	46/24	19.20 (7.96-46.31)^∗^	21/8	16.22 (4.65-56.59)^∗^	81/41	12.15 (4.38-33.75)^∗^
< 9.0	11/75	1.53 (0.58-4.01)	8/84	1.00 (ref.)	5/32	0.70 (0.23-2.11)	14/127	1.00 (ref.)
Test for interaction	*χ* ^2^ = 0.18 (1 df); *P* = 0.67				*χ* ^2^ = 0.01 (1 df); *P* = 0.93			
TG/HDL-C ratio								
≥ 4.2	57/20	21.56 (9.51-48.90)^∗^	43/24	13.30 (5.85-30.20)^∗^	20/5	24.83 (6.59-93.57)^∗^	80/39	12.88 (4.85-34.23)^∗^
< 4.2	10/80	0.91 (0.96-2.26)	11/84	1.00 (ref.)	6/35	0.74 (0.19-1.97)	15/129	1.00 (ref.)
Test for interaction	*χ* ^2^ = 0.97 (1 df); *P* = 0.33				*χ* ^2^ = 0.51 (1 df); *P* = 0.64			

^#^Indicated the numbers of subjects with and without MetS, respectively. ^†^Adjusted the effects of age and gender. OR: odds ratio; CI: confidence interval; TyG: triglyceride glucose; ref: reference. ^∗^The *P* value is < 0.01.

## Data Availability

The data that support the findings of this study are available on request from the author. The data are not publicly available due to privacy or ethical restrictions.
